# A Comparison between Bulgarian *Tanacetum parthenium* Essential Oil from Two Different Locations

**DOI:** 10.3390/molecules29091969

**Published:** 2024-04-25

**Authors:** Borislava Lechkova, Niko Benbassat, Diana Karcheva-Bahchevanska, Kalin Ivanov, Lyudmil Peychev, Zhivko Peychev, Stanislav Dyankov, Yoana Georgieva-Dimova, Krasimir Kraev, Stanislava Ivanova

**Affiliations:** 1 Department of Pharmacognosy and Pharmaceutical Chemistry, Faculty of Pharmacy, Medical University of Plovdiv, 4002 Plovdiv, Bulgaria; borislava.lechkova@mu-plovdiv.bg (B.L.); niko.benbasat@mu-plovdiv.bg (N.B.); diana.karcheva@mu-plovdiv.bg (D.K.-B.); kalin.ivanov@mu-plovdiv.bg (K.I.); stanislav.dyankov@mu-plovdiv.bg (S.D.); yoana.georgieva@mu-plovdiv.bg (Y.G.-D.); 2 Research Institute, Medical University of Plovdiv, 4002 Plovdiv, Bulgaria; 3 Department of Pharmacology, Toxicology and Pharmacotherapy, Faculty of Pharmacy, Medical University of Plovdiv, 4002 Plovdiv, Bulgaria; lyudmil.peychev@mu-plovdiv.bg; 4 Department of Medical Informatics, Biostatistics and E-Learning, Faculty of Public Health, Medical University of Plovdiv, 4002 Plovdiv, Bulgaria; zhivko.peychev@mu-plovdiv.bg; 5 Department of Propedeutics of Internal Diseases, Medical Faculty, Medical University of Plovdiv, 4002 Plovdiv, Bulgaria; kkraev@hotmail.com

**Keywords:** *Tanacetum parthenium*, feverfew, essential oil, GC/MS

## Abstract

*Tanacetum parthenium* L. (Asteraceae) is a perennial herbaceous plant with a long-standing historical use in traditional medicine. Recently *Tanacetum parthenium* L. essential oil has been associated with a promising potential for future applications in the pharmaceutical industry, in the cosmetics industry, and in agriculture. Investigations on the essential oil (EO) have indicated antimicrobial, antioxidant, and repellent activity. The present study aimed to evaluate the chemical composition of Bulgarian *T. parthenium* essential oil from two different regions, to compare the results to those reported previously in the literature, and to point out some of its future applications. The essential oils of the air-dried flowering aerial parts were obtained by hydrodistillation using a Clevenger-type apparatus. The chemical composition was evaluated using gas chromatography with mass spectrometry (GC-MS). It was established that the oxygenated monoterpenes were the predominant terpene class, followed by the monoterpene hydrocarbons. Significant qualitative and quantitative differences between both samples were revealed. Camphor (50.90%), camphene (16.12%), and bornyl acetate (6.05%) were the major constituents in the feverfew EO from the western Rhodope Mountains, while in the EO from the central Balkan mountains camphor (45.54%), trans-chrysanthenyl acetate (13.87%), and camphene (13.03%) were the most abundant components.

## 1. Introduction

*Tanacetum parthenium* L. (*T. parthenium*) is a perennial herbaceous plant belonging to the Asteraceae family with a long-standing historical use for traditional medicinal applications such as treatment of headache, fever, morning sickness, menstrual disorders, colic, skin conditions, arthritis, kidney pain, asthma etc. [1,2,3,4]. One of its common names is feverfew, presumably originating from its earlier name “featherfew” on account of its feather-like leaves [5]. It was reported that the prominent Greek physician Dioscorides recommended feverfew for “all hot inflammations” [1]. It is also suggested that the ancient Greeks called the herb “Parthenium,” because it was used as an important medicine for the treatment of workers who had fallen from the Parthenon during its construction in the 5th century AD [1]. The herb had an important role not only in ancient Greek traditional medicine but it was also used for the treatment of different disorders by the native peoples of Central and South America [1].

*Tanacetum parthenium* L. is a small, bushy, aromatic perennial plant. In general, its height is between 0.3 and 1 m [1]. The plant has yellow-green alternate leaves that are usually less than 8 cm in length. The leaves are described as almost hairless and chrysanthemum-like [1]. The diameter of its flowers is about 2 cm and they have a beautiful yellow colour. The flowers are arranged in a dense flat-topped cluster [1]. The bloom is from July to October. The flowers are sometimes compared to those of chamomile (*Matricaria chamomilla*), and both species could be confused for the other [1]. *Tanacetum parthenium* L. has a strong and bitter odour. Because of the similarities with chamomile, the plant is also known as wild chamomile, chamomile grande, grande chamomile, *Matricaria capensis, Matricaria eximia* hort, and *Matricaria parthenium* L. [1].

Other common names of *Tanacetum parthenium* L. are bachelor’s button, featherfoil, *Chrysanthemum parthenium*, altamisa, febrifuge plant, midsummer daisy, nosebleed, Santa Maria, wild quinine, chrysanthemum atricaire, federfoy, flirtwort, *Leucanthemum parthenium*, mother herb, *Parthenium hysterophorus*, parthenolide, *Pyrenthrum parthenium* L., feddygen fenyw, flirtroot, mutterkraut, and vetter-voo.

The species is indigenous to the Balkan Peninsula but is widespread in different areas all over the world, including in Europe, Asia, Australia, North Africa, and North America [1,6]. In the last decades, it has gained popularity as a remedy for migraine prophylaxis [7,8,9,10,11]. Recently, many efforts have been directed towards researching natural products with health-beneficial effects [12]. According to different studies, *T. parthenium* extracts exhibit anti-nociceptive [13,14,15], antioxidant [13,16,17,18,19,20,21], antibacterial [22], insecticidal [23], anti-inflammatory, neuromodulatory, antispasmodic, and uterine stimulant activity [24,25]. Investigations on feverfew EO have indicated antimicrobial [26,27,28,29,30,31], antioxidant [32], repellent [33], cytotoxic, and low anti-inflammatory [34] properties. Parthenolide, a sesquiterpene lactone isolated from the plant, has been reported to demonstrate anti-inflammatory [35,36,37,38,39,40,41,42,43,44,45], neuroprotective [46,47], cholesterol-lowering [48], antiviral [49], antileishmanial [50], and antitumor [51,52,53,54,55,56,57] effects. In a recent paper, Lakhera et al. suggested parthenolide as a candidate for the development of an anti-coronavirus drug [58]. *Tanacetum parthenium* contains various bioactive compounds including phenolic acids, flavonoids, coumarins, fatty acids, sesquiterpene lactones, and essential oil (EO) [4,59,60,61,62].

EOs are complex odoriferous mixtures of organic volatiles consisting of terpenes, terpenoids, phenylpropenes, and other compounds [63,64,65]. The number of individual constituents is usually around 20–60, but in some cases, it can even exceed 300 [66,67,68]. Generally, the main two or three that are present in higher concentrations are accountable for the biological activities of the EO. Nevertheless, those at minor concentrations could also affect the bioactivity by exhibiting additive effects, synergism, or antagonism [66,69,70]. Synergism occurs when the combined effect of multiple compounds is greater than the sum of their individual effects, while antagonism occurs when the combined effect is less than expected based on the individual effects of each compound. Understanding the complex interactions between the different compounds in the compositions of EOs is crucial for elucidating their biological activities and potential therapeutic applications. It also underscores the importance of considering the full chemical profile of EOs rather than focusing solely on individual compounds. Exploration of the full chemical profile of EOs plays an essential role in better understanding how EOs affect physiological processes and to potentially identify new therapeutic targets.

EOs exert various biological activities, such as anti-inflammatory, antimicrobial, and antioxidant, and have a multitude of applications [66,71,72], including the development of nutraceuticals and pharmaceuticals [73,74,75,76], cosmetic products (skin care, hair care, perfumery etc.) as active ingredients or as preservatives [71], and in aromatherapy [77,78]. As antibiotic resistance is becoming a major concern in modern medicine, EOs and their components have been widely investigated for antimicrobial effects alone or in combinations [79,80]. Furthermore, they are used in the food industry as flavouring agents, preservatives, and food packaging materials [75,81,82,83]. Recently, EOs have been associated with a promising potential for utilisation as alternatives for synthetic pesticides in agriculture [84,85].

The chemical composition of EOs varies depending on plant origin, plant organ, development stage, edaphic and climatic factors, as well as the method of extraction, drying method, etc. [29,66,86,87,88]. Literature data regarding the genus *Tanacetum* reveals that a significant variation in terms of EO constituents and chemovariability is observed on the species and subspecies levels [89]. Studies on the relationship between phytochemicals produced by plant metabolism and their effects are especially important for exploring the drug-discovery potential of the plant species [90,91].

The present study aimed to evaluate the chemical composition of Bulgarian *T. parthenium* EOs from two different locations and to expand the knowledge of its quantitative and qualitative differences, which could serve as a starting point in the selection of plant material for further cultivar development. Moreover, the results were compared with EOs from other geographical regions, highlighting some future application perspectives.

## 2. Results

**Figure 1:**
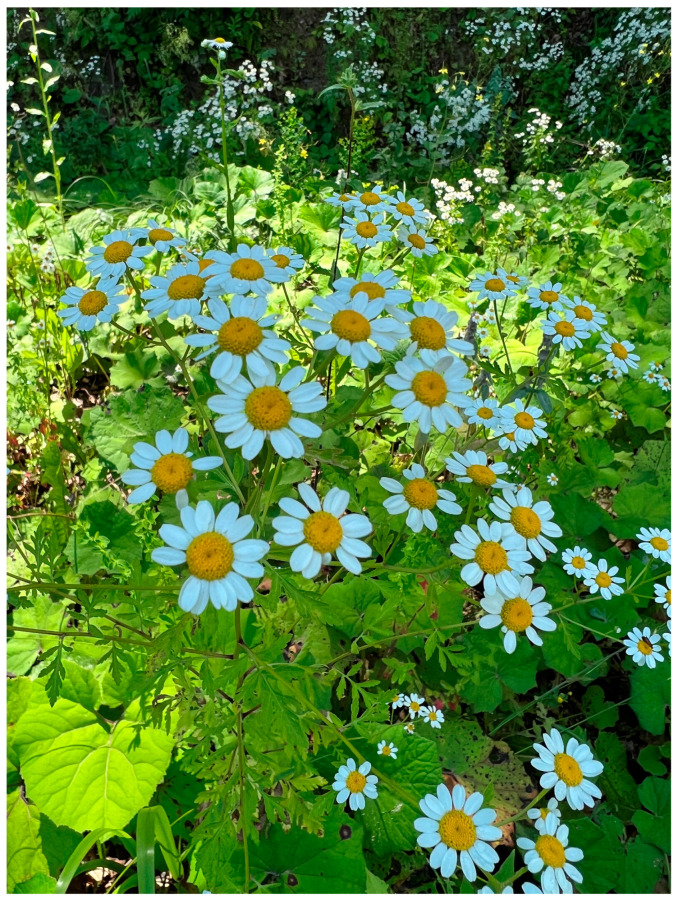
Wild-growing populations of *T. parthenium* L.

The flowers of wild-grown *T. parthenium* L. were collected from two different mountain regions in Bulgaria: Tsigov Chark (41°56′23.2″ N 24°11′17.4″ E), western Rhodope Mountains (RM), and Gabrovo (42°49′39.7″ N 25°18′17.6″ E), central Balkan Mountains (BM) (Figure 1). The two locations differ in altitude, soil type, and climate (Table 1).

**Figure 2:**
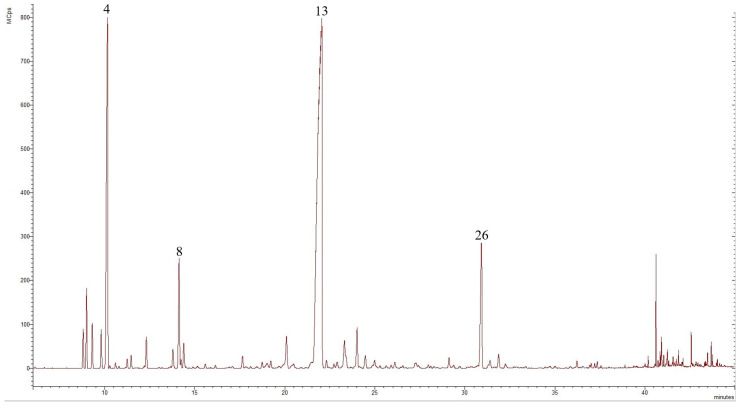
Chromatogram of *T. parthenium* EO from the wild population in the western Rhodope Mountains, compounds derived from GC-MS analysis, in which the numbers refer to the following: **4**—camphene, **8**—p-cymene, **13**—camphor, and **26**—bornyl acetate.

**Figure 3:**
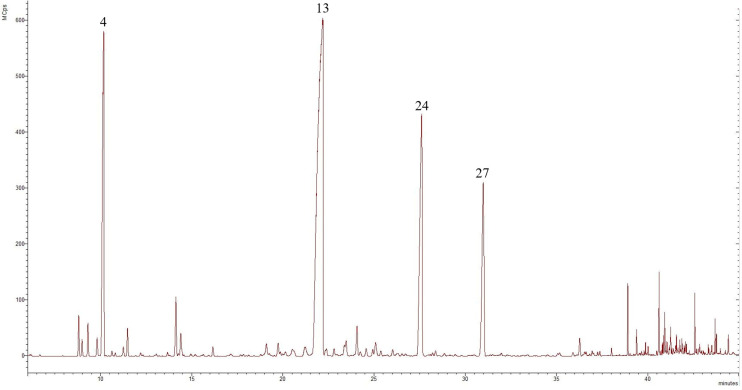
Chromatogram of *T. parthenium* EO from the wild population in the central Balkan Mountains, compounds derived from GC-MS analysis, in which the numbers refer to the following: **4**—camphene, **13**—camphor, **24**—trans-chrysanthenyl acetate, and **27**—trans-verbenyl acetate.

Both obtained EOs were pale yellow in colour, with a distinct aroma. Their chemical composition was analysed by GC-MS, which resulted in the identification of 25 volatile components in the EO from the wild feverfew population in RM, representing 90.52% of the total oil, while 28 volatile compounds were detected in the EO extracted from plants collected in BM, representing 91.41% of the total oil. The predominant class terpenes in both samples were the oxygenated monoterpenes, accounting for 63.77% (RM sample) and 70.93% (BM sample), followed by the monoterpene hydrocarbons, representing 26.03% and 18.52%, respectively. The content of sesquiterpene hydrocarbons and other compounds was minimal, ranging between 0.05–1.33% and 0.63–0.67%, respectively, and oxygenated sesquiterpenes were absent in both EOs. The chromatograms of the EOs from wild-grown *T. parthenium* collected in the western Rhodope Mountains and the central Balkan Mountains are presented in Figure 2 and Figure 3, respectively.

The GC-MS analysis revealed some significant differences in the volatile constituents of the two EOs. Table 2 shows the chemical composition of the EOs with formulas, retention indices, class terpenes, and relative percentage amounts. Twenty-five different compounds were established in the EO obtained from plants from RM and 28 compounds in the EO from plants collected in BM. The oxygenated monoterpenes were the most abundant constituents in both of the samples (>60%), followed by the monoterpene hydrocarbons (26.03% in the RM EO and 18.52% in the BM EO).

## 3. Discussion

The main compound found in both samples was camphor, in concentrations between 45.54% and 50.90%. The camphene content was also similar (13.03–16.12%).

Camphor is a bicyclic monoterpene ketone that can be obtained from plants or can be produced synthetically, the difference being that natural camphor is dextrorotatory and synthetic camphor is optically inactive [96]. Major natural sources of this compound include the wood of *Cinnamomum camphora* L. (camphor tree, camphor laurel) (Lauraceae family) and the leaves from *Ocimum kilimandscharicum* Gürke (also known as camphor basil) (Lamiaceae family) [96,97]. Camphor is also a constituent of the EOs of several aromatic plant species such as *Cinnamomum agasthyamalayanum*, *Ocimum canum*, *Salvia officnalis*, *Rosmarinus officinalis*, *Lavandula* sp., *Artemisia* sp. etc. [98,99,100,101,102]. Camphor has a long-standing use as an antiseptic, antipruritic, abortifacient, aphrodisiac, counterirritant and rubefacient, heart stimulant, fumigant, and a fragrance and flavoring agent [96,103,104]. It has also been used to relieve nasal congestion, pain, and inflammation [96,105,106]. Furthermore, camphor exhibited neuroprotective effects [107], insecticidal activity [108], and skin penetration enhancing properties [109]. In addition, it can be utilised in the synthesis of new important molecules [104,106]. Despite these beneficial actions, the potential risks associated with the use of camphor and camphor containing products should not be overlooked, since there are many reports of intoxication due to irrational use, especially in children [96,110,111,112]. However, products with this substance are generally considered safe for topical application as long as the indications and dosage are followed [96].

Camphene belongs to the group of monoterpene hydrocarbons; it is present in the EOs of various aromatic plants, fruits, and spices and has been used in the food and cosmetics industries as a flavouring substance or fragrance ingredient [113,114]. It was reported to inhibit ROS generation, NO release, and decrease lipid peroxidation showing cytoprotective and antioxidant activity [114]. Quintans-Júnior et al. also reported high free radicals scavenging activity and strong antioxidant effect as well as modest antinociceptive activity [115]. In addition, camphene attenuates muscle atrophy by inhibiting oxidative stress [116]. Moreover, camphene demonstrates hypolipidemic [117,118], anti-inflammatory [119,120], anti-tumor [121], anti-hepatosteatotic [122], and insecticidal [123,124,125] effects. Camphene-based derivatives have shown to be promising agents against pathogens, such as *Mycobacterium tuberculosis*, *Staphylococcus aureus*, *Enterococcus* spp., and different viruses [126,127,128].

Despite the similarity in the content of the two main compounds, there are some important differences in the composition of the EO isolated from the plant material from Balkan Mountain and the plant material from Rhodope Mountains. Trans-Chrysanthenyl acetate was found only in the EO from *Tanacetum parthenium* L. growing in the central Balkan Mountains (13.87%). The presence or the absence of chrysanthenyl acetate in the different samples could affect the biological activity of the EO. Recently, it has been reported that chrysanthenyl acetate has an indirect antioxidant activity, increasing the activity of antioxidant enzymes [129]. Apart from members of the *Tanacetum* genus [130,131,132,133,134], it also occurs in high concentrations in species such as *Anthemis maritime* [135], *Lamium amplexicalule* [136], *Zieria cytisoides* [137], and *Allium neapolitanum* [138]. A content of 100% (E)-chrysanthenyl acetate was reported in oil obtained from *Anthemis secundiramea* Biv. subsp. *secundiramea* flowers [129]. The high amount of chrysanthenyl acetate is associated with potent phytotoxic, antioxidant, and antimicrobial activity [139,140].

Trans-Verbenyl acetate is another compound that is presented only in the composition of the EO from *Tanacetum parthenium* L. growing in the central Balkan Mountains. Trans-Verbenyl acetate was previously detected in feverfew EO obtained by steam distillation but the content was only 0.5% [141]. It has also been found in other members of the *Tanacetum* genus in relatively low concentrations [142,143]. The content of this compound in the BM sample was significant (8.93%). Trans-Verbenyl acetate belongs to the class of the oxygenated monoterpenes; however, data on its effects are very scarce. Nishino et al. reported on sex pheromonal activity in a study using the American cockroach (*Periplaneta americana* L.) [144].

Although the composition of the EO isolated from the plant material from the central Balkan Mountains was more abundant, bornyl acetate was found only in the sample from the Rhodope Mountains (6.05%). It is a bicyclic monoterpene with promising anti-inflammatory and immunomodulatory effects and low toxicity [145]. Due to its anti-oxidant and anti-inflammatory properties, it has been suggested as a potential therapeutic agent in the treatment of atherosclerosis [146], osteoarthritis [147], autoimmune demyelinating diseases (including multiple sclerosis) [148], and memory disorders [149]. Several studies pinpointed its insecticidal effects and possible use as an environmentally friendly biopesticide [124,125,150]. Inhalation of bornyl acetate in low doses causes a sedative effect without affecting vigilance [151]. Intravenous administration of this compound leads to vasorelaxation [152]. Furthermore, it exhibits analgesic [153,154], anti-proliferative [155,156], and anti-abortive [157] effects.

P-cymene was detected in both EOs, however the concentration in the RM sample was more notable (3.89%). It is an aromatic monoterpene with a distinctive woody, spicy scent, found in over 200 foods and spices, including cinnamon, nutmeg, carrots, raspberries, orange juice, grapefruit, tangerine, and is a major compound in EOs from members of the *Thymus*, *Origanum*, *Ocimum*, *Artemisia*, *Protium*, *Eucalyptus*, *Hyptis*, and *Zataria* genus with a plethora of health beneficial properties [158,159,160,161,162,163,164,165]. P-cymene demonstrates anti-inflammatory and anti-nociceptive effects [115,159,160,162,166,167]. Including it in a complex with β-cyclodextrin could even improve the analgesic and anti-inflammatory properties [163]. Additionally, p-cymene exhibits anti-oxidant activity and could be used as a neuroprotective agent [168]. The anti-oxidant and anti-inflammatory potential could attribute to the gastroprotective effect of the substance revealed in an ethanol-induced gastric ulcer in rats [169]. Moreover, p-cymene can prevent beta-amyloid-caused synaptic plasticity impairment in a rat model of Alzheimer’s disease [170] and is reported to possess vasorelaxant [171,172] and anti-tumor effects [161], the latter being mainly manifested when p-cymene is associated with metals in complexes such as ruthenium [173] and osmium [174]. As a natural antimicrobial component, p-cymene in low concentrations could increase the shelf life of un-pasteurised fruit juices [175]. It enhances the antimicrobial properties of other components and exerts an anti-biofilm activity [176].

In a study on tansy EO composition, Nurzyńska-Wierdak et al. assumed less environmental influence on camphor content compared to genetic factors [177]. A higher concentration was noted in plants in acidic sites. On the contrary, more trans-chrysanthenyl acetate was detected in plants from a location with alkaline and neutral soils [177]. Thus, a less acidic pH of the soil in BM could explain the lower amount of camphor and the higher share of trans-chrysanthenyl acetate compared to the RM sample. Estell et al. reported a positive effect of UV light restriction on camphene, bornyl acetate, and p-cymene content [178]. The more significant percentage of p-cymene in the RM sample could be linked to the altitude [179].

*Tanacetum parthenium* EO analyses have been conducted by several authors. Table 3 compares the main constituents of feverfew EOs obtained from several different geographical regions.

Camphor has been recognised as the main compound identified in feverfew EOs from various locations, including Turkey, Egypt, Iran, Serbia, Italy, and Tajikistan. Typically, its content is around 45–65% [6,25,26,27,28,30,131,180,181,182,183], which is in agreement with our results. Sharopov et al. detected a concentration of up to 94% in EO from a wild population growing in Tajikistan [34]. Végh et al. determined the highest amount of camphor in the leaves of the plant during flowering [184]. However, considerable variations have been documented concerning the other predominant components. Trans-Chrysanthenyl acetate was noted as the second most abundant constituent in EOs extracted from leaves, flowers, and aerial parts in concentrations ranging from 21.12% to 33.8% [25,27]. In contrast, in other studies it was not detected at all [6,19,26,29,34,180,181,183] or was found in relatively small amounts/traces [27,30]. Camphene emerged as another characteristic compound of feverfew EO, with a fraction reaching up to 13.74% [6,19,25,26,27,28,29,30,34,131,180,181,182]. The present study supports these data, and the RM sample afforded an even higher camphene amount (16.12%). On the contrary, this substance was lacking in EO from *T. parthenium* cultivated in Northern Italy [183]. Unlike previous results, Giuliani et al. also reported a large content of farnesol (28.83%) belonging to the oxygenated sesquiterpenes [183], which were completely absent in this investigation. The amount of sesquiterpene hydrocarbons in the RM and BM samples was negligible (only 0.05 and 1.33%, respectively). In contrast to the Bulgarian feverfew EOs, the share of SH in the EO from a wild sample of *T. parthenium* from Iran was relatively large (14.9%), the major representative from this class being germacrene-D (9.2%) [181]. Shafaghat et al. detected SH as the second most abundant class of terpenes (after MO), at 16.1%, in EO from feverfew leaves, represented by considerable content of trans-β-farnesene (8.3%) and β-caryophyllene (5.9%) [180]. In regards to bornyl acetate, the data vary as well, from its total lack [25,27,183] to 18.35% detected in the EO from *T. parthenium* collected during flowering in Iran [29]. Mohsenzadeh et al. suggested a correlation between the concentration of the compounds and the developmental stage [29]; however, other factors could also affect the composition of EOs- geographical area, plant material, environmental factors, methods used for drying and extraction, etc. [6,27,182,185]. Shahhoseini et al. concluded a concentration dependent positive effect on the quantity and quality of feverfew EO by titanium dioxide-nanoparticles application [186].

The chemical profiles of both analysed EOs indicate a prominent potential for implementation in environmentally friendly pest control products, which are gaining importance since synthetic pesticides are subject to more and more restrictions due to their negative impact on human health and the environment [187]. The obtained results suggest the two *T. parthenium* EOs as suitable bioresources for incorporation in products for external application (creams, ointments, gels, patches, etc.) aimed at relieving joint and muscle pain, especially in patients with chronic inflammatory disorders (such as osteoarthritis and rheumatoid arthritis) since the use of synthetic drugs (for instance non-steroidal anti-inflammatory drugs) for long periods of time is associated with an increased risk of severe side effects, even when applied topically. Further in vitro and in vivo investigations are needed to evaluate these activities and to clarify the feverfew EO utilisation perspectives.

## 4. Materials and Methods

### 4.1. Plant Materials

The flowers of wild-grown *T. parthenium* L. were collected from two locations in Bulgaria: Tsigov Chark (41°56′23.2″ N 24°11′17.4″ E), western Rhodope Mountains, and Gabrovo (42°49′39.7″ N 25°18′17.6″ E), central Balkan Mountains. The plants were authenticated by Associate Professor Niko Benbassat in accordance with the European Pharmacopoeia [188]. Voucher specimens (No. 063400 from RM and No. 063395 from BM) were deposited in the Herbarium of the University of Agriculture, Plovdiv, Bulgaria. The two locations differ in altitude (RM 1100 m, BM 500 m), soil type (RM Dystric Cambisols, BM Haplic Luvisols), and climate (RM Middle Mountain region, BM Temperate Continental region) [92]. The plant material was collected in the phase of full flowering and was then dried at room temperature.

### 4.2. Chemicals and Reagents

For the determination of the retention indices (RI) of the separated compounds, the following hydrocarbons were used: nonane (≥99%), decane (≥99%), undecane (≥99%), dodecane (99%), tridecane (≥99%), tetradecane (≥99%), and hexadecane (≥99%) purchased from Merck KGaA (Darmstadt, Germany). Hexane (Thermo Fisher Scientific GmbH, Bremen, Germany) was used for the dilution of the EO.

### 4.3. Isolation of the Essential Oil

The essential oils of the air-dried flowering aerial parts were obtained by hydrodistillation for 4 h using a Clevenger-type apparatus. The collected essential oils were dried over anhydrous sodium sulfate and stored in dark glass vials at 4 °C until GC-MS analysis.

### 4.4. Chromatographic Conditions

The analysis of both EOs was carried out using gas chromatography with mass spectrometry (GC-MS). For the analysis, a Bruker Scion 436-GC SQ MS (Bremen, Germany)equipped with a Zebron ZB-5MSplus capillary column (0.25 µm film thickness and 30 m × 0.25 mm i.d.) was used. The carrier gas was helium with a constant flow rate of 1 mL/min. The volume of the injection was 1 µL, with the temperature of the injector set to 250 °C and split ratio of 1:20. The oven temperature was initially set at 50 °C for 1 min, then increased to 130 °C at a rate of 2 °C/min, and then increased to 240 °C at a rate of 15 °C/min and held for 1 min. The detector temperature was set to 300 °C. The mass spectra were collected in a full scan mode with a mass range of 50–350 *m*/*z*. The retention indices (RI) of the separated compounds were calculated from the retention times of the C8–C30 n-alkane series injected under the same conditions described above. The identification of the essential oil constituents was achieved by comparing their MS spectra and RI values with spectral data within the Wiley NIST11 Mass Spectral Library (NIST11/2011/EPA/NIH) and the literature data. The analyses were performed in triplicate. Standard deviations (SDs) did not exceed 2% of the obtained values of each component.

## 5. Conclusions

Studies on the phytochemicals produced by plant metabolism have significant importance for exploring the drug-discovery potential of the plant species. The present study focused on a comparative evaluation of the chemical composition of *T. parthenium* EO from two locations in Bulgaria. The occurrence of two chemotypes based on the predominant components—camphor/camphene/bornyl acetate chemotype (RM) and camphor/trans-chrysanthenyl acetate/camphene chemotype (BM) was established. Although there are some similarities in the composition, the two samples are characterised by important differences. For targeting some future applications and performing biological activity studies, it is important to consider the full chemical profile of EOs rather than focusing solely on individual compounds. Understanding the possible interactions between the different compounds found in the compositions of EOs is crucial for elucidating their biological activities and potential therapeutic applications. Exploration of the full chemical profile of EOs is essential for better understanding how EOs affect various physiological processes and potentially identify new therapeutic targets.

## Figures and Tables

**Table 1:** Comparison between the plant collection locations.

Region	Altitude	Climate	Soil Type	Main Soil Characteristics	Ref.
RM	1100 m	Middle Mountain region Average annual temperature: 5–10 °C. Mean annual precipitation: 600–800 mm.	Dystric Cambisols	Rich in humus (up to 12%). pH 5.5–6. Colour: brown to reddish.	[92,93]
BM	500 m	Temperate Continental Region Average annual temperature: 10–11 °C. Mean annual precipitation: 750–800 mm.	Haplic Luvisols	Poor in humus (2–3%). Poor water permeability. pH: acidic. Poor in Nitrogen and Phosphorus. The clay content increases with the depth of the soil (13% in 2–10 cm depth, 18% in 10–20 cm depth). The sand content is about 20%.	[92,94,95]

**Table 2:** Volatile organic compounds in essential oil obtained from wild populations of *Tanacetum parthenium* L. growing in the western Rhodope Mountains (RM) and in the central Balkan Mountains (BM), where “–‘’ = not detected.

No	Compound	RI	Formula	Class of Compound	% of Total RM	% of Total BM
**1**	Tricyclene	924	C_10_H_16_	MH	1.06	0.85
**2**	α-Thujene	927	C_10_H_16_	MH	2.14	0.35
**3**	α-Pinene	932	C_10_H_16_	MH	1.24	0.72
**4**	Camphene	947	C_10_H_16_	MH	16.12	13.03
**5**	Benzaldehyde	954	C_7_H_6_O	O	0.17	0.06
**6**	Sabinene	965	C_10_H_16_	MH	0.26	0.20
**7**	β-Pinene	968	C_10_H_16_	MH	0.39	0.66
**8**	p-Cymene	1012	C_10_H_14_	MH	3.89	1.63
**9**	d-Limonene	1015	C_10_H_16_	MH	0.93	0.82
**10**	γ-Terpinene	1045	C_10_H_16_	MH	–	0.26
**11**	Chrysanthenone	1110	C_10_H_14_O	MO	1.33	–
**12**	trans-p-Mentha-2,8-dienol	1116	C_10_H_16_O	MO	–	0.37
**13**	Camphor	1137	C_10_H_16_O	MO	50.90	45.54
**14**	1-(1,4-dimethyl-3-cyclohexen-1-yl)-ethanone	1146	C_10_H_16_O	MO	–	0.09
**15**	exo-2,7,7-trimethylbicyclo[2.2.1]heptan-2-ol	1147	C_10_H_18_O	MO	–	0.21
**16**	cis-Sabinol	1162	C_10_H_16_O	MO	0.71	–
**17**	Terpinen-4-ol	1174	C_10_H_18_O	MO	1.55	0.52
**18**	p-Cymen-8-ol	1181	C_10_H_14_O	MO	0.58	–
**19**	trans-p-Mentha-1(7),8-dien-2-ol	1183	C_10_H_16_O	MO	–	0.26
**20**	cis-Myrtenal	1188	C_10_H_14_O	MO	–	0.21
**21**	α-Terpineol	1191	C_10_H_18_O	MO	0.19	0.59
**22**	trans-Piperitol	1204	C_10_H_18_O	MO	0.12	0.18
**23**	trans-Carveol	1208	C_10_H_16_O	MO	0.28	–
**24**	trans-Chrysanthenyl acetate	1232	C_12_H_18_O_2_	MO	-	13.87
**25**	Cuminaldehyde	1238	C_10_H_12_O	MO	0.10	–
**26**	Bornyl acetate	1286	C_12_H_20_O_2_	MO	6.05	–
**27**	trans-Verbenyl acetate	1287	C_12_H_18_O_2_	MO	–	8.93
**28**	Carvacrol	1294	C_10_H_14_O	MO	0.48	–
**29**	Myrtanol acetate	1385	C_12_H_20_O_2_	MO	–	0.16
**30**	(Z)-Jasmone	1390	C_11_H_16_O	O	0.09	0.08
**31**	Caryophyllene	1402	C_15_H_24_	SH	–	0.13
**32**	(E)-β-Farnesene	1417	C_15_H_24_	SH	0.05	0.89
**33**	β-Copaene	1425	C_15_H_24_	SH	–	0.31
**34**	1,7,7-Trimethylbicyclo[2.2.1]heptan-2-yl (E)-2-methylbut-2-enoate	1445	C_15_H_24_O_2_	MO	1.48	–
**35**	Z-spiroether	1782	C_13_H_12_O_2_	O	0.27	0.31
**36**	E-spiroether	1791	C_13_H_12_O_2_	O	0.14	0.18
	Terpene classes					
	Monoterpene hydrocarbons (MH)				26.03	18.52
	Oxygenated monoterpenes (MO)				63.77	70.93
	Sesquiterpene hydrocarbons (SH)				0.05	1.33
	Oxygenated sesquiterpenes (SO)				-	-
	Others (O)				0.67	0.63
	Total identified				90.52	91.41

The percentage of relative peak area is the average value of three measurements. The standard error of the mean was eliminated, and it was not more than 2%.

**Table 3:** Comparison of the main volatile compounds *of T. parthenium* EO from different geographical areas.

Region	Plant Material	Main Volatile Compounds	Other Volatile Compounds	Ref.
Bulgaria (RM)	Flowers	camphor (50.90%), camphene (16.12%), bornyl acetate (6.05%)	p-cymene (3.89%)	*
Bulgaria (BM)	Flowers	camphor (45.54%), trans-chrysanthenyl acetate (13.87%), camphene (13.03%)	trans-verbenyl acetate (8.93%)	*
Turkey	Aerial parts	camphor (56.9%), camphene (12.7%), p-cymene (5.2%)	bornyl acetate (4.6%), chrysanthenone (2.5%)	[6]
Egypt	Flowers	camphor (48.4%), E-chrysanthenyl acetate (26.3%), camphene (8.76%)	bornyl angelate (1.81%), thymol (1.81%)	[25]
Egypt	Leaves	camphor (37.7%), E-chrysanthenyl acetate (33.8%)	terpin-1-ol (5.14%), camphene (3.72%), terpinene (3.1%), bornyl angelate (2.17%)	[25]
Iran	Leaves	camphor (53.8%), trans-β-farnesene (8.3%), camphene (6.9%), β-caryophyllene (5.9%)	4-hydroxy-benzenepropanoic acid (3.7%), chrysanthenone (3.3%), bornyl acetate (3.1%), borneol (2.9%)	[180]
Iran	Aerial parts (wild)	camphor (50.5%), germacrene-D (9.2%), camphene (7.7%)	E-sesquilavandulol (4.8%), E-myrtanol (4.7%)	[181]
Iran	Aerial parts (cultivated)	camphor (57.6%), E-chrysanthenyl acetate (25.1%)	camphene (4.6%), bornyl angelate (2.2%)	[181]
Iran	Flowers (shade-dried)	camphor (49.3%), chrysanthenyl acetate (25.8%), camphene (11.2%)	α-pinene (3.3%), bornyl acetate (1.6%), β-pinene (1.1%)	[182]
Iran	Flowers (sun-dried)	camphor (48.5%), chrysanthenyl acetate (25.4%), camphene (11.0%), verbenone (9.0%), α-terpineol (8.0%), α-phellandrene (5.5%)	α-pinene (3.0%), p-cymene (1.8%), bornyl acetate (1.5%), β-pinene (1.1%)	[182]
Iran	Flowers (oven-dried)	camphor (47.5%), chrysanthenyl acetate (23.8%), camphene (9.9%)	α-pinene (4.1%), bornyl acetate (1.5%), β-pinene (1.1%), limonene (0.8%)	[182]
Iran	Flowers	camphor (61.1%), camphene (9.2%)	farnesol (4.6%), bornyl acetate (3.5%), chrysanthenon (3.1%), borneol (2.9%)	[26]
Serbia	Aerial parts (cultivated, different seed origin)	camphor (46.4–47.2%), trans-chrysanthenyl acetate (22.4–27.3%), camphene (10.9–12.7%)	bornyl acetate (2.3–3.2%), p-cymene (1.6–2.9%), α-pinene (1.2–2.1%)	[131]
Iran (Hamedan)	Aerial parts, stem/leaf, inflorescence	camphor (11.61–53.39%), trans-chrysanthenyl acetate (8.85–22.54%), camphene (5.11–10.45%)	p-cymene (4.15–4.18%), α-pinene (0.1–2.55%), bornyl acetate (0.48–2.05%)	[27]
Iran (Tehran)	Aerial parts, stem/leaf, inflorescence	camphor (11.52–52.98%), trans-chrysanthenyl acetate (7.63–22.28%), camphene (5.46–10.26%)	limonene (0.89–1.04%)	[27]
Iran	Aerial parts (three developmental stages)	camphor (12.65–18.94%), bornyl acetate (11.48–18.35%), camphene (9.5–13.74%), borneol (8.7–11.84%), juniper camphor (4.71–6.23%)	δ-cadinene (2.86–4.25%), bornyl isovalerate (2.26–3.26%), β-eudesmol (1.96–2.65%), p-cymene (1.96–2.29%)	[29]
Turkey (Davutpasa- Istanbul)	Aerial parts	camphor (49%), trans-chrysanthenyl acetate (22.1%), camphene (9.4%)	bornyl acetate (2.9%), p-cymene (1%)	[30]
Turkey (Savsat- Ardahan)	Aerial parts	camphor (60.8%), camphene (6.8%)	chrysanthenone (3.2%), bornyl acetate (3.7%), p-cymene (1.9%)	[30]
Iran	Aerial parts	camphor (45%), chrysanthenyl acetate (21.5%), camphene (9.6%)	p-cymene (4.15%), α-pinene (3.55%), bornyl acetate (2.88%)	[28]
Tajikistan	Aerial parts	camphor (69.7–94.0%), camphene (1.7–12.2%), bornyl acetate (4.2–8.7%)	β-farnesene (0–2.9%), germacrene D (0–1.9%)	[34]
Italy	Aerial parts	camphor (56.83%), farnesol (28.83%)	caryophylladienol (2.19%)	[183]
Iran	Aerial parts	camphor (27.75–29.1%), neryl acetate (8.94–11.05%), p-cymene (5.93–7.01%)	bornyl acetate (4.02–5.94%), neo-intermedeol (3.93–4.23%), camphene (3.45–4.01%)	[19]

* Present study.

## Data Availability

Data are contained within the article.
